# Development of a high-precision evaluation system for radial pulse wave applanation tonometry devices

**DOI:** 10.1038/s41598-026-45661-4

**Published:** 2026-03-31

**Authors:** Min-Ho Jun, Seunghwan Choy, Young-Min Kim

**Affiliations:** 1 Digital Health Research Division, Korea Institute of Oriental Medicine (KIOM), Daejeon, 34054 Republic of Korea; 2 R&D Strategy Division, Korea Institute of Oriental Medicine (KIOM), 1672 Yuseongdaero, Yuseong-gu, Daejeon, 34054 Republic of Korea

**Keywords:** Pulse wave evaluation system, Artificial radial artery, Precise pulse simulator, Applanation tonometry devices, Radial artery pulse wave, Cardiology, Engineering, Health care, Medical research

## Abstract

The demand for wearable devices for daily health management and tonometry-based pulse wave measurement has grown rapidly. However, the absence of standardized and physiologically realistic evaluation systems remains a major barrier to developing reliable and clinically applicable devices. Although ISO 18615:2020 defines general performance requirements for electronic radial pulse tonometric devices, no evaluation platform has been available to assess these standards with higher precision. We developed a novel high-precision evaluation system that integrates a 3D cam-based pulse wave generation unit, wrist simulation unit, system control unit, and applied pressure unit. This platform enables controlled and reproducible simulation of physiological pulse waves, including variations in base pressure, pulse pressure, applied pressure, and radial skin and arterial properties, which have been difficult to replicate in previous systems. The system was designed not only to meet but also to exceed ISO 18,615 requirements, thereby enabling precise performance assessment. Validation experiments evaluated pulse wave precision and range, as well as artificial radial artery characteristics. The system demonstrated high reproducibility and accuracy. The mean coefficients of variation (CVs) were 0.29% for applied pressure, 0.11% for pulse waves, and 0.05% for pulse rate. The CVs of pulse wave shape parameters H and T were 1.06% and 1.60%, respectively. The systole/diastole ratio (SDR) ranged from 24.45% to 57.69% with a 1.04% error ratio, the pulse rate from 19.93 bpm to 204.90 bpm with a 0.34% error ratio, and the base pressure from − 3.17 mmHg to 116.51 mmHg with a 3.60% error ratio. This study presents the first comprehensive evaluation platform capable of reproducing physiologically realistic radial arterial pulse waves with high precision and reproducibility. By referencing and surpassing ISO 18,615 performance requirements, the system provides rigorous and reliable assessment of device performance. This platform reduces variability, enables precise calibration, and offers a benchmark for evaluating tonometry-based and wearable pulse wave measurement devices. It is expected to accelerate the development of high-quality cardiovascular monitoring technologies and support the clinical translation of wearable health monitoring devices.

**Supplementary Information:**

The online version contains supplementary material available at 10.1038/s41598-026-45661-4.

## Introduction

Pulse wave measurement devices are primarily used for diagnosing cardiovascular diseases such as arteriosclerosis^1,2^, coronary artery disease^3^, and hypertension^4^ and are also used to monitor cardiovascular status during surgery^5^. The most accurate method of measuring pulse waves is through invasive catheter-based techniques; however, owing to the limitations of invasive procedures, such as the risk of complications and infections, noninvasive methods have been developed. Recently, wearable devices have been developed to manage cardiovascular health in everyday life outside specialized medical facilities. These noninvasive techniques can be broadly classified into two categories: tonometry^6^, which records the radial artery pulse wave via a pressure sensor, and photoplethysmography (PPG)^7^, which detects changes in blood volume corresponding to heartbeats via a photodiode sensor.

In tonometry-based pulse wave measurement devices, pressure is applied to the skin to measure the pulse wave of the radial artery located beneath the skin. When using this method, the measurement results can be influenced by the characteristics of the skin and blood vessels. Consequently, simulators need to provide an environment that closely mimics real measurement situations, not only by replicating simple pressure but also by considering the properties of the intermediate medium between the vessel and the measurement sensor. Additionally, according to the recent international standard ISO 18615:2020 regarding the general requirements of electric radial pulse tonometric devices, they must have a certain level of accuracy in terms of applied pressure, pulse pressure, pulse rate, and measurement position, and these parameters can be measured via a pulse waveform simulator. However, very few devices capable of evaluating tonometry-based pulse wave measurement devices exist.

The previously developed pulse simulators are generally classified into three main types: a nonvascular simulator that uses linear motors to replicate pulsations^8,9^, a cam-based simulator that generates pulsatile fluid in a simulated vessel by pressing a mock vessel with a cam^10,11^, and a piston-based simulator that creates pulsatile fluid in a simulated vessel via a piston‒cylinder system^12^. While cam-based and piston-based simulators can generate waveforms that more closely resemble pulse waves via simulated vessels, further research is needed to develop simulators that account for intermediate medium characteristics, such as blood vessels or skin. Additionally, to evaluate tonometry-based pulse wave measurement devices, it is necessary to assess the applied pressure level. However, there has been little research on the development of devices for comprehensive evaluation, including pulse pressure, base pressure, applied pressure, pulse shape, and pulse rate. Therefore, further research in this area is needed.

While previous simulators have contributed to waveform generation, they exhibit several critical limitations, including the inability to replicate intermediate tissue properties, limited control and measurement of applied pressure, restricted range and precision of pulse pressures, and lack of comprehensive evaluation capabilities. These constraints limit their applicability for evaluating tonometry-based pulse wave measurement devices. To address these gaps, we developed a high-precision evaluation system integrating a 3D cam-based pulse generator, applied pressure motor, dual-volume control system, and artificial skin and vessel modules. This system directly addresses the need for a simulator that combines physiological realism with precise and reproducible evaluation metrics, enabling more accurate and reliable assessment of tonometry devices.

## Methods

We developed an integrated evaluation system specifically for tonometry-based pulse wave measurement devices with two primary objectives: (1) to overcome the technical limitations of previous simulators by providing physiologically realistic and reproducible testing conditions, and (2) to establish a versatile platform applicable to both device evaluation and research in cardiovascular monitoring and wearable healthcare. The international standard ISO 18615:2020 defines the general performance requirements for electric radial pulse tonometric devices. With reference to this standard, we designed our system to achieve higher precision and broader control than the device-level requirements. According to ISO 18,615, the specified performance criteria include an applied pressure (AP) range of 0–120 mmHg with a resolution of 2 mmHg and accuracy of ± 6 mmHg (± 5%), a pulse pressure (PP) range of 0–105 mmHg with a resolution of 1 mmHg and accuracy of ± 5 mmHg (± 5%), and a pulse rate (PR) range of 40–150 bpm with a resolution of 1 bpm and accuracy of ± 5 bpm. Pulse pressure (PP) is defined as the difference between systolic blood pressure (SBP) and diastolic blood pressure (DBP), representing the pressure amplitude during a single cardiac cycle (PP = SBP − DBP). By aligning with and surpassing these requirements, the developed system ensures both technical rigor and clinical relevance, serving as a robust platform for evaluating tonometry-based pulse wave measurement devices.

### Development of an evaluation system for pulse wave measurement devices

**Fig. 1:**
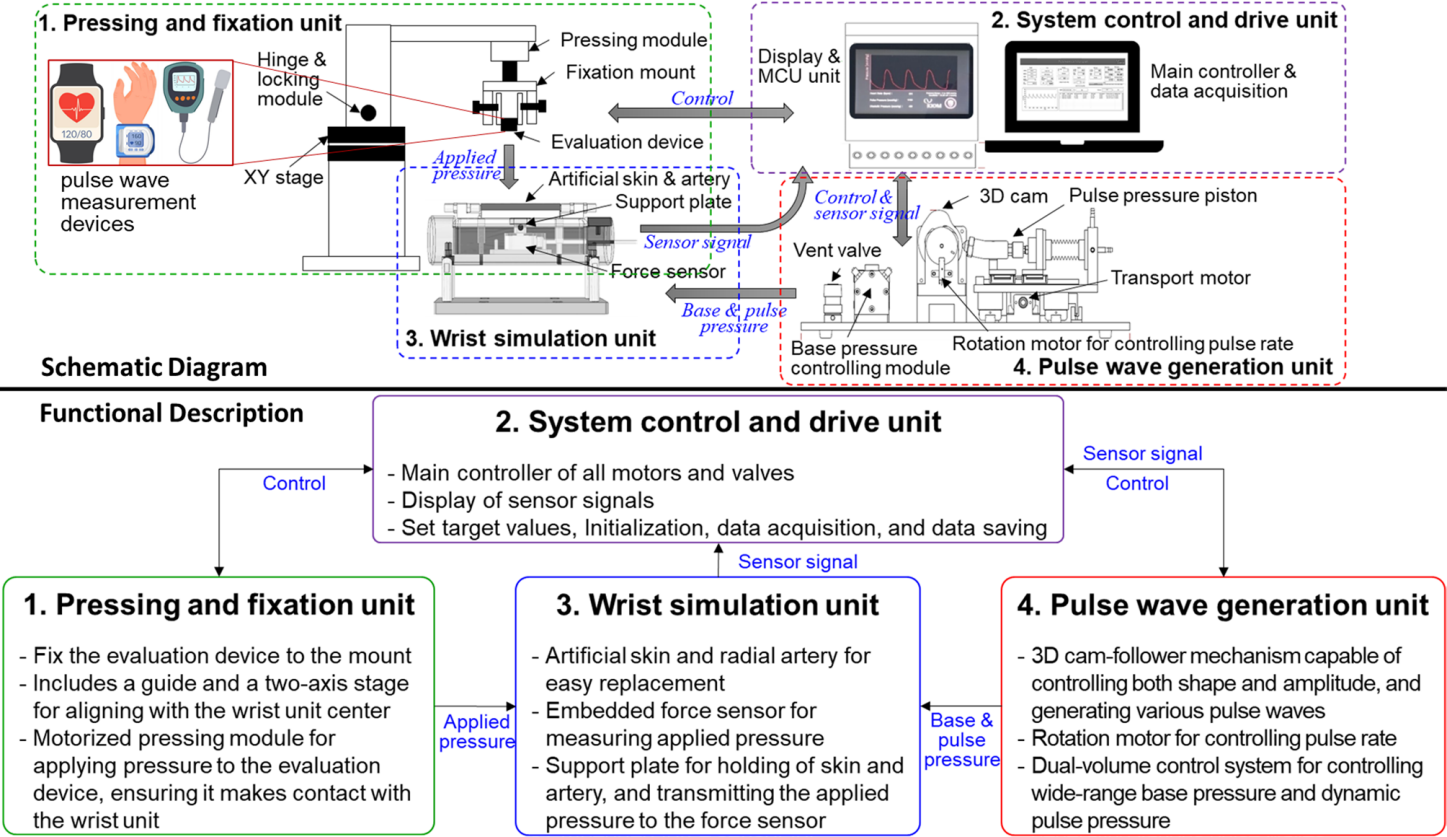
Conceptual diagram of the evaluation system for tonometry pulse wave measurement devices and functional representation of the units within the system.

**Fig. 2:**
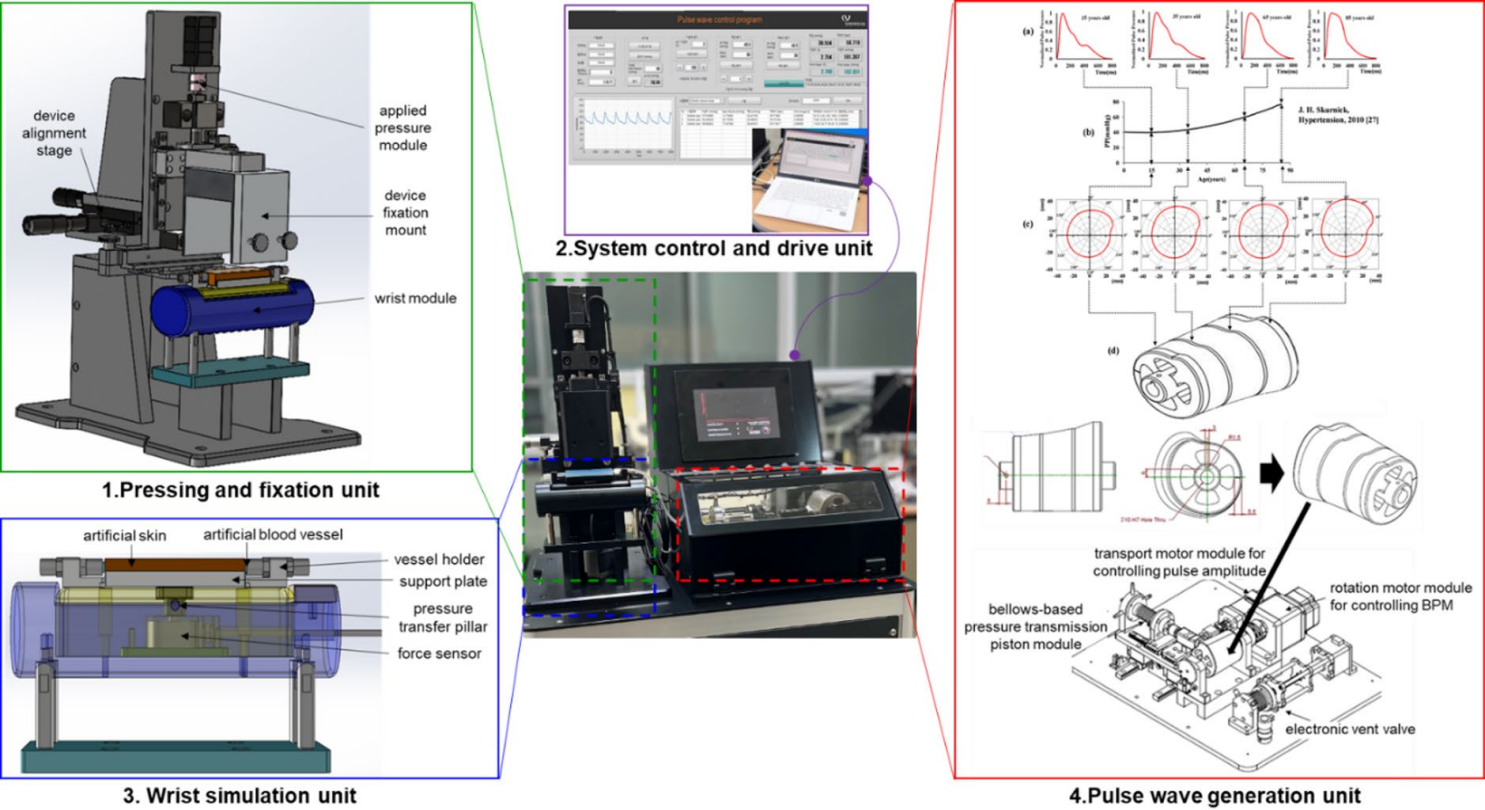
Main components of an evaluation system for the tonometry pulse wave measurement devices; (1) a pressing and fixation unit, (2) a system control and drive unit, (3) a wrist simulation unit, and (4) a pulse wave generation unit.

To develop an evaluation system for evaluating tonometry-based pulse wave measurement devices, a pulse wave simulator was designed using a 3D cam-follower mechanism. This system consists of (1) a pressing and fixation unit, (2) a system control and drive unit, (3) a wrist simulation unit, and (4) a pulse wave generation unit to estimate the pulse wave measurement device. The developed evaluation system is based on a previously designed 3D cam-based pulse wave simulator^10–13^ and has been enhanced with several key features: a fixation and alignment module for securing the measurement device in a pressing and fixation unit, a precise applied pressure module in a pressing and fixation unit, signal analysis algorithms implemented in a system control and drive unit, an interchangeable artificial skin and vessel module that mimics human skin and blood vessels in a wrist simulation unit, and a dual-volume control unit for wider and more precise pulse wave reproduction in a pulse wave generation unit. Figure 1 shows the conceptual diagram of the evaluation system for tonometry pulse wave measurement devices. It depicts the mounting of the evaluation device on the pressing and fixation unit, with base pressure and pulse pressure generated by the pulse wave generation unit sent to the wrist simulation unit to produce pulse waves. The device is then pressed onto the artificial radial artery and skin, resembling the human body, to evaluate the performance of the tonometry pulse wave measurement device. Figure 2 shows the main components and the developed evaluation system for the tonometry pulse wave tube to the artificial skin layer of the wrist simulation unit, enabling the evaluation of pulse wave measurement devices in measurement devices, which includes (1) the pressing and fixation unit, (2) the system control and drive unit, (3) the wrist simulation unit, and (4) the pulse wave generation unit The radial pulse wave generated by the cam-based pulse wave generation unit is transmitted through an artificial blood vessel a manner that closely resembles actual measurements on the human body.

**Fig. 3:**
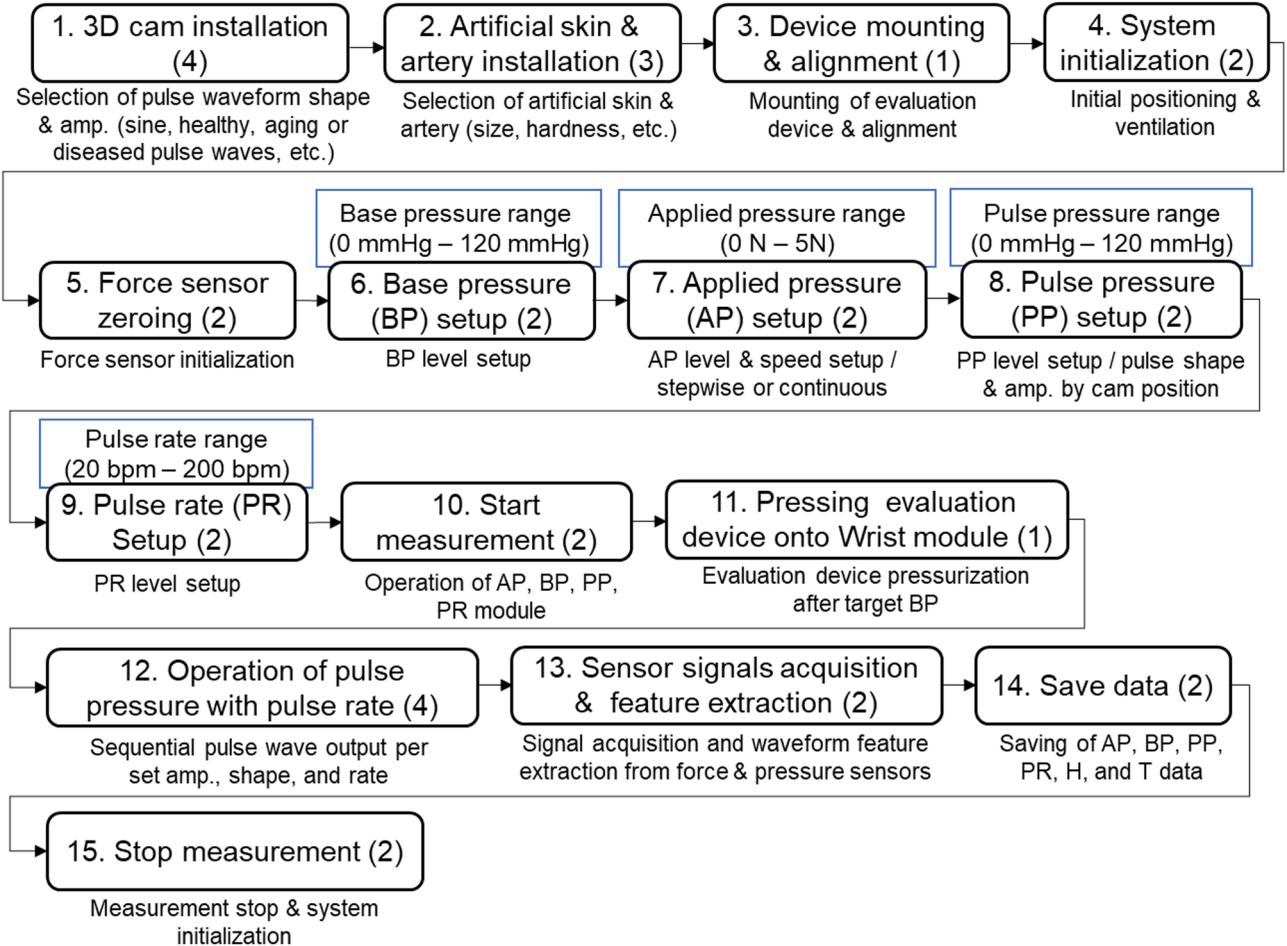
Operational procedure of the evaluation system for the tonometry pulse waveform measurement devices.

Figure 3 illustrates the step-by-step procedure for operating the evaluation system for tonometry pulse wave measurement devices. The process begins with the installation of a 3D cam (Step 1), followed by the attachment of the artificial artery and skin onto the wrist module (Step 2). After mounting and fixing the evaluation device in its designated position (Step 3), the system is initialized (Step 4), and the force sensor is zeroed to ensure baseline accuracy (Step 5). Subsequently, three core pressure parameters are configured: base pressure (BP, Step 6), applied pressure (AP, Step 7), and pulse pressure (PP, Step 8). The pulse rate (PR) is then set to simulate different heart rates (Step 9). Once the contact area of the evaluation device’s sensor on the wrist simulation unit is entered, the system control program automatically converts the applied pressure from newtons (N) to millimeters of mercury (mmHg). After all values are configured, measurement is initiated (Step 10), and upon reaching the target BP, the evaluation device is pressed onto the wrist module (Step 11). In Step 12, the simulator operates by synchronously generating pulse waveforms through regulated pulse pressures at the set pulse rate. Sensor signals, including force and pressure data, are acquired and processed to extract key features (Step 13). Finally, the collected data is saved (Step 14), and the measurement process is concluded (Step 15). Some steps in the procedure may be repeated according to the measurement context or testing protocol. This structured sequence allows for precise control and repeatability in evaluating the performance of wearable tonometry devices under various physiological simulation conditions.

### Component modules of an evaluation system for pulse wave measurement devices

First, (1) the pressing and fixation unit for the pulse wave measurement device includes a guide and two-axis positioning plate for device mounting and sensor alignment, a sensor housing jig to fix various types of pulse wave measurement devices, a precision pressing motor, and a 90-degree tilting hinge for easy attachment and detachment of the measurement devices. After the measurement sensor or device is fixed to the device fixation mount and attached to the pressing and fixation unit, the alignment guide is used to align the measurement axis by positioning the artificial artery and the measurement sensor or device. Once the base pressure is set, pressure is applied to the artificial artery until the set value is reached. When the contact button on the system control and drive unit is pressed, the measurement sensor or device attached to the pressing and fixation unit receives feedback from the force sensor implanted in the wrist simulation unit, and the sensor stops when it makes contact with the artificial skin (at 0.02 N; adjustable). In this state, the applied pressure, pulse pressure, pulse rate, and other parameters are set, and sensor evaluation is performed. The applied pressure is gradually increased to the set value at the programmed speed (adjustable) and maintained. To ensure accurate and precise maintenance of the applied pressure, feedback control from the force sensor allows for control of the initial motor speed and dependent speed before reaching the set applied pressure.

**Fig. 4:**
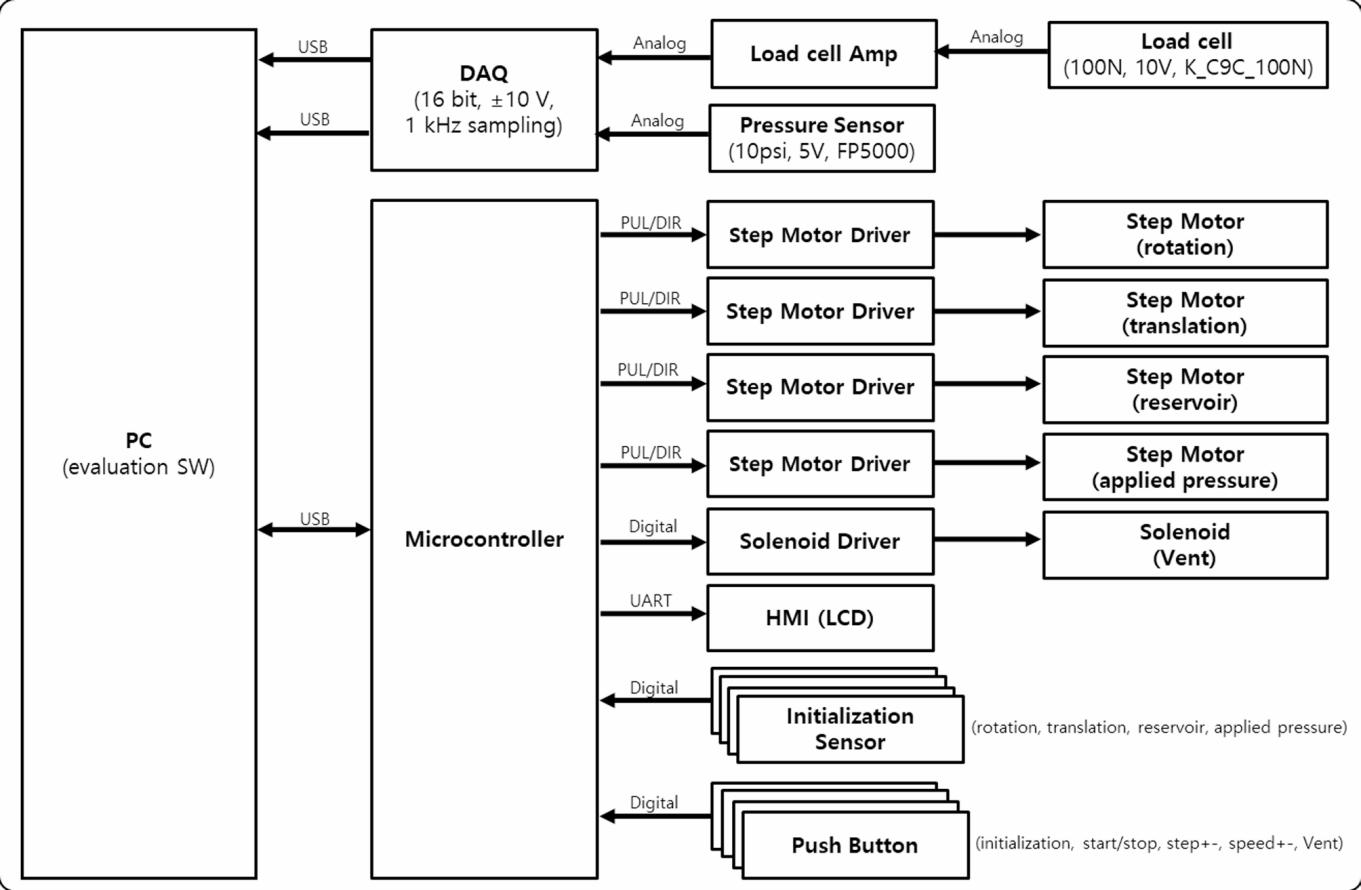
System block diagram of the evaluation system for the tonometry pulse wave measurement device.

**Fig. 5:**
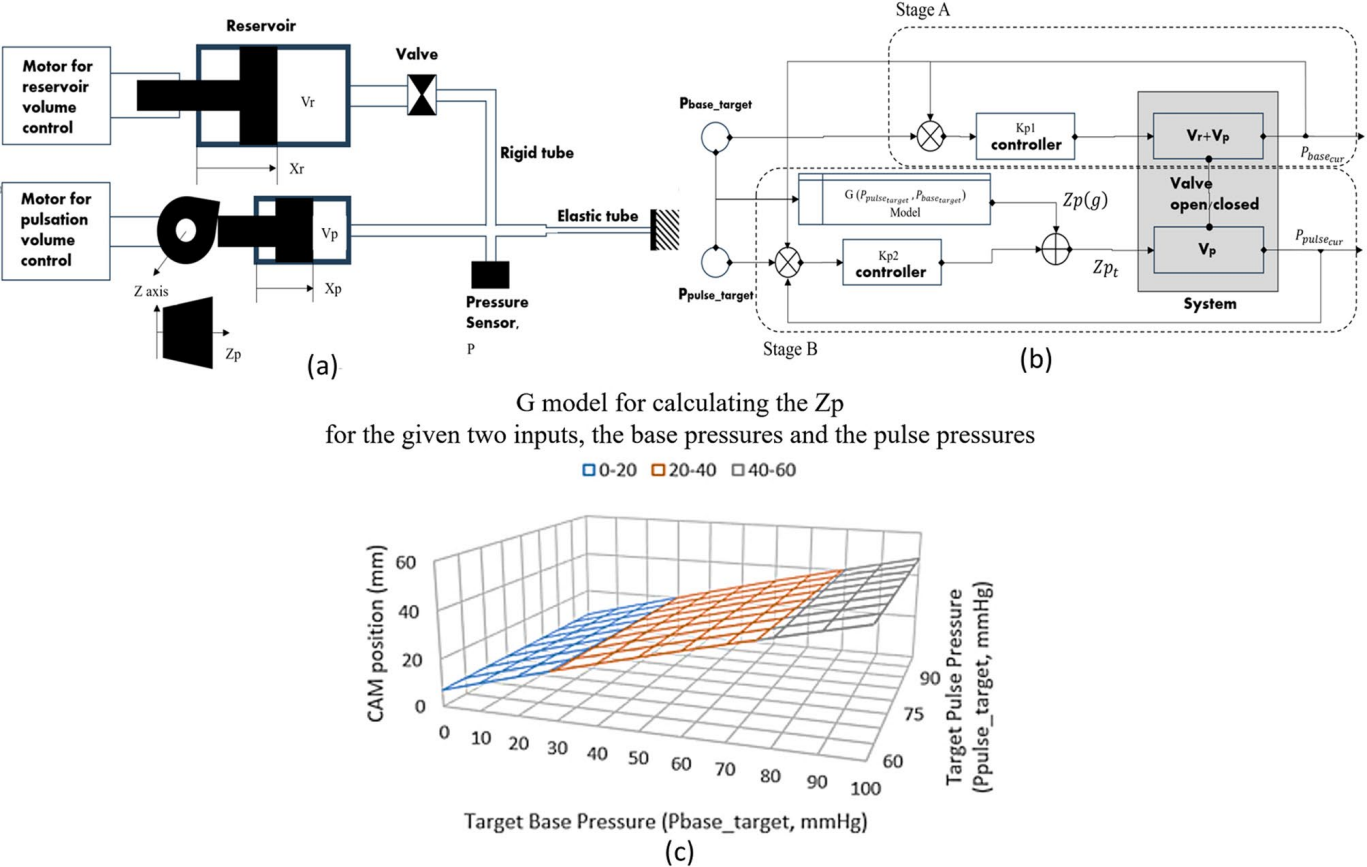
Conceptual diagram (**a**), algorithm (**b**) of the dual-volume control system with a check valve for maintaining wide-range stable static pressure and achieving precise and dynamic pulse pressure control, and G model (**c**) for determining the z-axis position of the 3D cam. * Vr is the reservoir volume with respect to the base pressure, Vp is the pulsation volume with respect to the pulse pressure, Xr and Xp are the stroke lengths of the piston to adjust the Vr and Vp, Zp is the horizontal position by the z-axis translation of the 3D cam, Xp is determined by the combination of the z-axis translational movement and the rotational movement of the 3D cam, and Kpi is the proportional gain for the i-th P controller.

(2) The system control and drive unit is built around a LabVIEW-based main controller, featuring USB communication between the embedded MCU unit—responsible for motor and valve control—and the sensor signal processing board for precise measurement. Sensors for the precise measurement of applied pressure and pulse pressure convert signals through an NI DAQ into 16-bit digital values within a ± 10 V range, which are transmitted to the main program at a 1 kHz sampling rate. Step motors and drive controllers for pressure application, cam rotation, and vascular pressure control are simultaneously managed through the MCU. The initialization sensors for the motors, pressure control sensors, and system operation buttons send digital input signals directly to the MCU. A block diagram of the evaluation system for the pulse wave measurement devices is shown in Fig. 4. Sensor data are collected via a data acquisition (DAQ) system, and the simulator is controlled through a microcontroller. The entire system is managed through USB communication with a PC, enabling centralized control and monitoring.

(3) The wrist simulation unit is designed to replicate the human wrist, comprising structures that represent the radius bone, radial artery, and skin layers (dermis and epidermis). A force sensor is utilized to measure the applied pressure of the tonometry pulse wave measurement device. This unit also includes a support plate for easy attachment and detachment of the artificial skin and vessel, a vessel holder to fix the artificial artery, and a pressure transfer pillar for effective transmission of applied pressure to the force sensor.

(4) The pulse wave generation unit, improved from previous studies^10,11,13,14^, utilizes a 3D cam to control both the shape and amplitude of the pulse wave. Moving the cam horizontally can linearly increase the amplitude and reproduce various pulse wave shapes. Additionally, the pulse rate can be controlled by adjusting the rotation speed of the cam. When one full rotation of the cam generates one pulse wave cycle, the cam’s rotations per minute correspond to the beats per minute (bpm) of the pulse. This pulse wave generation unit builds on a previously developed pulse wave simulator model with the addition of a dual-volume control system for enhanced pulse pressure modulation. The pulse wave simulator requires independent adjustment of the base pressure, which corresponds to the diastolic blood pressure, and pulse pressure, which represents the amplitude of the pulse wave. The base pressure needs a wide control range (0 mmHg − 100 mmHg) with stable static pressure maintenance, while the pulse pressure must be precisely and dynamically controlled. To achieve this, a check valve and a control algorithm were added to develop the dual- volume control system, as shown in Fig. 5. In this system, the total reservoir volume (Vr) including the second reservoir volume (Vp) and the connecting tube volumes regulates the base pressure by the reservoir volume motor system. When the base pressure reaches the set value, the check valve closes, and the Vp, which is controlled by the position (*Zp*) of the cam and has a relatively much smaller volume, more dynamically adjusts the pulse pressure magnitude in wide ranges, as shown in Fig. 5a. This configuration enables the development of a precise dual-volume control system. The base pressure control system reliably converges to the target values because of the large volume (Vr + Vp) control. After the check valve is closed, the target cam position (*Zp(g)*) is determined by the G model, which is designed by modulating the cam pulse wave data designed for 80 mmHg base pressure according to the target base pressure values and the target pulse pressure values. Figure 5c shows the three-dimensional plot of the G model, in which the desired cam position values are almost linearly interpolated for the given two inputs, the pulse pressure and the base pressure. Figure 5b shows the control schematic diagram of the proportional gain control system. At the stage A, *Kp*_*1*_ controller is firstly used to control the base pressure. The *Kp*-specific control results of the base pressure and pulse pressure magnitudes, obtained from the proposed dual-volume control system, is presented in Supplementary Figure S1. When the current values reach for the set values within the given error bounds, the stage B simultaneously controls both the baseline pressure and pulse pressure magnitude by feeding the errors of the current pulse and base pressure values with the following *Kp*_*2*_ control Eq. (1)

1 $$\:Z{p}_{t}={Zp\left(g\right)}_{0}+{Kp}_{2}\left\{\left({P}_{puls{e}_{target}}-{P}_{puls{e}_{cur}}\right)+\left({P}_{bas{e}_{target}}-{P}_{bas{e}_{cur}}\right)\right\}$$

where,

$\:Z{p}_{t}$: the horizontal position of the 3D cam at time t.

$\:Z{p\left(g\right)}_{0}$: the target horizontal position of the 3D cam initially determined by the G model.

$\:{P}_{puls{e}_{target}}$: the target pulse pressure.

$\:{P}_{bas{e}_{target}}$: the target base pressure.

$\:{P}_{puls{e}_{cur}}$ : the current pulse pressure measured at time t.

$\:{P}_{bas{e}_{cur}}$ : the current base pressure measured at time t.

and proper tuning of the proportional gain helps stabilize pressure control.

By properly tuning *Kp*_*2*_, the steady-state errors converge to average error bounds of 0.41 mmHg for base pressures and 0.56 mmHg for pulse pressure magnitudes, as summarized in Table 1.

**Table 1:** Experimental results for the steady-state errors (SSEs) of the base pressure (BP) and pulse pressure (PP) with the dual volume control system (KP_1_=KP_2_ =10).

Target Pressures (mmHg)	SSE	
	BP (mmHg)	PP (mmHg)
40.00	0.31	0.38
60.00	0.36	0.41
80.00	0.45	0.63
100.00	0.52	0.83
mean	0.41	0.56
SD	0.08	0.18

## Experimental assessments

**Fig. 6:**
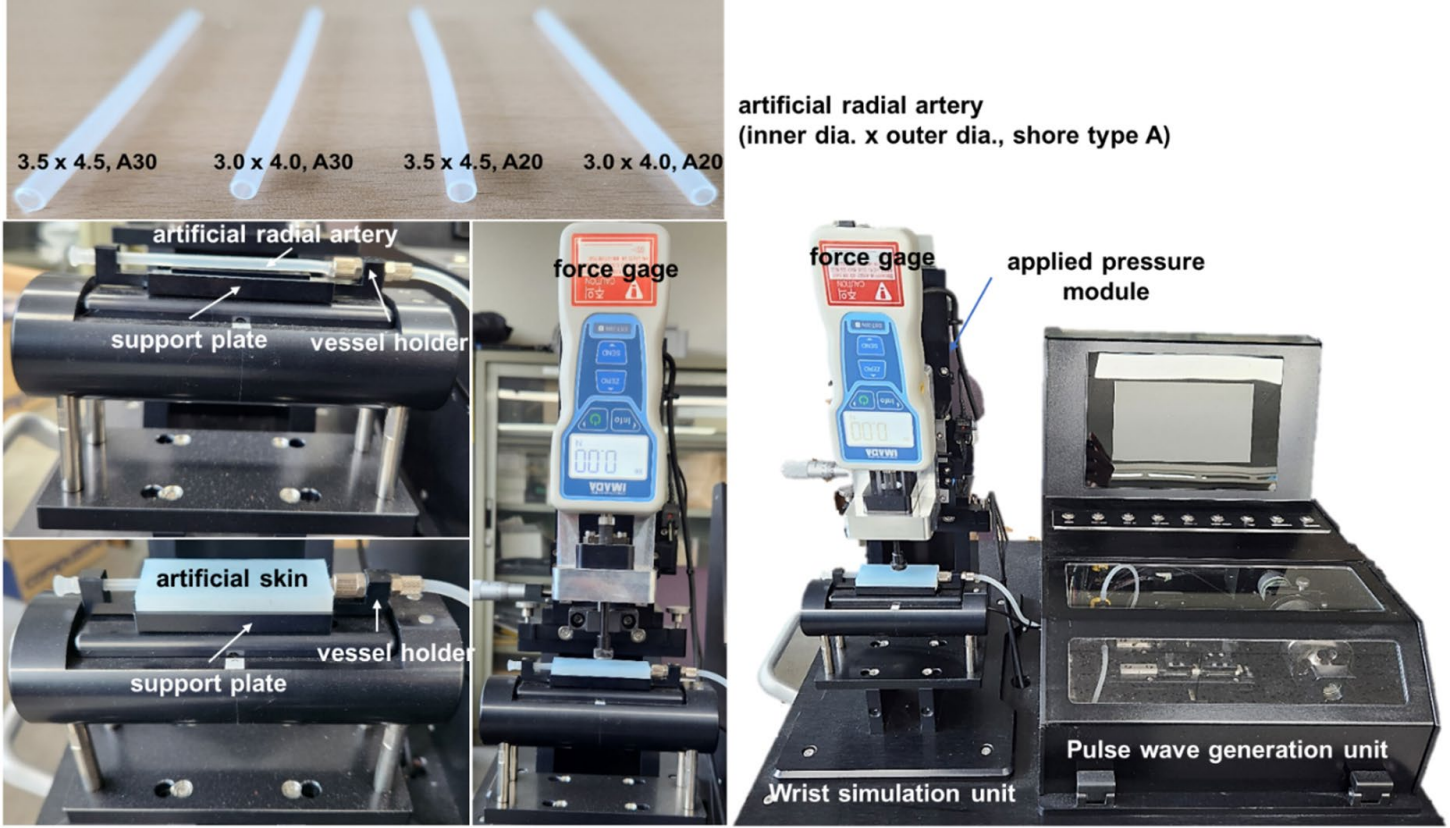
Experimental setup to evaluate the performance of the developed system and assess the effect of artificial radial artery size and stiffness.

To evaluate the performance of the developed evaluation system for pulse wave measurement devices, experiments were conducted to assess the systole/diastole ratio range, pulse rate range, diastolic pressure range, applied pressure precision, pulse pressure precision, pulse rate precision, and pulse wave shape precision. Additionally, to evaluate the effect of radial artery characteristic changes on pulse wave measurement, artificial radial arteries of different sizes and stiffnesses were fabricated and used in the experiments. The experimental setup for evaluating the performance of the developed system is shown in Fig. 6.

To evaluate the systole/diastole ratio range, the base pressure of the developed system was fixed at 60 mmHg, the applied pressure was 2 N, and the pulse rate was 60 bpm. The pulse pressure magnitude was adjusted to 20 mmHg, 40 mmHg, 60 mmHg, and 80 mmHg, the data were recorded, and the system performance was analyzed. The systolic value is the sum of the base pressure and the pulse pressure, whereas the diastolic value is equal to the base pressure. The systole/diastole ratio (SDR) is calculated by subtracting the diastolic value from the systolic value, dividing by the systolic value, and multiplying by 100. The formula is as follows:

2 $${\mathrm{SDR}}=\left( {\left( {{\mathrm{Systole}} - {\mathrm{Diastole}}} \right)/{\mathrm{Systole}}} \right) \times {\mathrm{1}}00$$

The SDR is an indicator that reflects the pulse pressure relative to the base pressure. Through this ratio range, the variation range in pulse pressure can be determined. For the pulse rate range, the pulse pressure was fixed at 40 mmHg, the base pressure was 60 mmHg, and the applied pressure was 2 N. The pulse rate was adjusted to 20 bpm, 75 bpm, 130 bpm, and 205 bpm, the data were recorded, and the system performance was evaluated. To measure the diastolic pressure range, the pulse pressure was fixed at 40 mmHg, the pulse rate was 60 bpm, and the applied pressure was 2 N. The base pressure was adjusted to 0 mmHg, 40 mmHg, 80 mmHg, and 120 mmHg, the data were recorded, and system performance was evaluated. To evaluate the precision of the applied pressure, the base pressure was fixed at 60 mmHg, and the applied pressure was adjusted to 1 N, 2 N, 3 N, 4 N, and 5 N. Measurements were repeated 20 times for each setting, and the mean and standard deviation were calculated. A certified push‒pull gauge (DST-20 N, Imada, Japan) was installed on the pressing and fixation unit to measure the applied pressure precision. To measure the precision of the pulse pressure, the base pressure was fixed at 60 mmHg, the applied pressure was 2 N, and the pulse rate was 60 bpm. The pulse pressure was adjusted to 40 mmHg, 60 mmHg, 80 mmHg, and 105 mmHg, with measurements repeated 20 times for each setting. The means and standard deviations were then calculated. To assess the precision of the pulse rate, the base pressure was fixed at 60 mmHg, the applied pressure was 2 N, and the pulse pressure was 40 mmHg. The pulse rate was adjusted to 50 bpm, 75 bpm, 100 bpm, and 125 bpm, with measurements repeated 20 times for each setting. The mean and standard deviation were calculated to evaluate the pulse rate precision. The precision of the pulse wave shape was evaluated via data obtained from pulse pressure precision experiments.

**Fig. 7:**
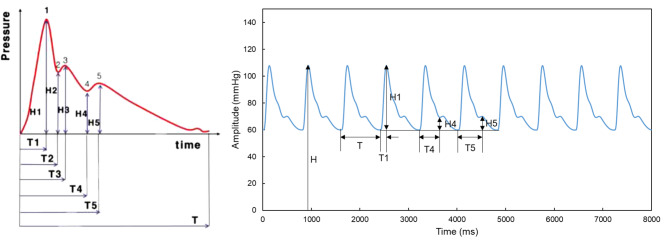
The left graph represents the feature points in the pulse wave (ISO18615:2020), ranging from H1 to H5 and T1 to T5 and the right graph displays the feature points extracted from the developed system.

From the obtained pulse shape, the first peak amplitude (H1) and its arrival time (T1), the second valley amplitude (H4) and its arrival time (T4), the third peak amplitude (H5) and its arrival time (T5), and the sum of the base pressure and the pulse pressure (H) and the pulse cycle time (T) were derived. H2, H3, T2, and T3 were excluded from the analysis of pulse shape precision because these features could not be extracted from the experimental pulse waves, as shown in Fig. 7. The parameters H1/H, H4/H, H5/H, T1/T, T4/T, and T5/T were extracted and analyzed. For each parameter, 20 repeated measurements were taken, and the mean and standard deviation were calculated to assess the pulse wave shape precision. To assess the performance of the pressure sensor (FP5000, Honeywell, USA) utilized in the developed evaluation system, a reference pressure monitoring device (RPM4, Fluke, USA) was employed to verify its validity. To evaluate whether the performance of the developed evaluation system differs depending on the size and stiffness of the artificial blood vessel, experiments were conducted using a validated push–pull gauge. Artificial vessels were fabricated with two inner diameters (3.0 mm and 3.5 mm) and a thickness of 0.5 mm, while two stiffness levels (Shore A 20 and A 30) were tested. The artificial skin, made from Dragon Skin™ 20 and 30 (Smooth-On, Inc., PA, USA), was kept constant in size and structure throughout the experiments to maintain consistent surface contact conditions. The experimental results showed a distinct decrease in pulse wave amplitude when the applied pressure exceeded a certain threshold, indicating an oscillometric response consistent with arterial compression characteristics. These measurements were obtained under continuous pressure application rather than stepwise loading.

To analyze the influence of arterial size and stiffness on measurement performance, only data within the range below the applied pressure threshold were processed. The pulse wave signals were filtered using a low-pass filter, and peak values were extracted to determine waveform periodicity and identify individual cycles, as shown in Supplementary Figure S2. Each waveform was segmented based on the detected peaks, its DC offset removed, and the peak values were sequentially plotted to visualize waveform progression. From these processed data, key parameters—including the maximum pulse pressure (PP), applied pressure magnitude, and the slope of pulse pressure increase—were derived and analyzed to assess the device’s response under varying arterial conditions.

## Results

The experimental results revealed that the systole/diastole ratio ranged from 24.4% to 57.6%, the pulse rate ranged from 19.9 bpm to 203.9 bpm, and the diastolic pressure ranged from − 3.2 mmHg to 116.5 mmHg. In the pulse wave precision evaluation experiment, the standard deviation (SD) of the applied pressure (AP) was ± 0.510 mmHg, the SD of the pulse pressure (PP) was ± 0.069 mmHg, and the SD of the pulse rate (PR) was ± 0.040 bpm. Additionally, the precision of the pulse wave shape was characterized by SDs of ± 0.002 for amplitude rate (H) and ± 0.006 for time rate (T). The precision of the applied pressure was evaluated through five repeated measurements, which revealed a range of 68.7 mmHg to 338.3 mmHg within the applied force range of 1 N to 5 N for the force gauge. The applied pressure varies depending on the contact area of the sensor of the pulse wave measurement device. Once the sensor’s contact area is entered into the control program, the applied force (N) is automatically converted into pressure (mmHg). Pulse pressure (PP) and base pressure (BP) are directly obtained from the calibrated pressure sensor, which outputs pressure values in mmHg. The results for arterial property evaluation are measured using a calibrated force gauge, and the output signals are expressed in force (N) to represent vessel deformation characteristics. The results demonstrated an average standard deviation of 0.510 mmHg, indicating precise control of the applied pressure. Additionally, a variation of approximately 0.29% from the set applied pressure was observed. Pulse pressure precision was calculated by obtaining the standard deviation from data collected through 20 repeated experiments for each set pulse pressure. The results revealed an average standard deviation of 0.069 mmHg, indicating a variation of approximately 0.11% from the pulse pressure. The precision of the pulse rate was evaluated by analyzing the results of 20 repeated measurements at each target value, yielding an average standard deviation of approximately 0.040 bpm. This indicates a variation of 0.05% from the pulse rate. The precision of the pulse shape was evaluated through 20 repeated experiments for each pulse pressure. Ratios of H and T values were calculated, and their averages and standard deviations were determined.

**Table 2:** Experimental results for pulse wave precision.

Target precision for applied pressure (AP), pulse pressure (PP), and pulse rate (PR)												
No	Applied pressure (AP)				Pulse pressure (PP)				Pulse rate (PR)			
	Force gauge (N)	Mean (mmHg)	SD (mmHg)	CV (%)	Target (mmHg)	Mean (mmHg)	SD (mmHg)	CV (%)	Target (bpm)	Mean (bpm)	SD (bpm)	CV (%)
1	0.998	68.666	0.323	0.470	40.000	40.024	0.069	0.173	50.000	49.785	0.048	49.785
2	1.999	137.519	0.287	0.209	60.000	60.017	0.067	0.111	75.000	74.655	0.030	74.655
3	3.005	203.675	0.822	0.403	80.000	79.995	0.047	0.058	100.000	99.536	0.049	99.536
4	4.006	271.201	0.673	0.248	105.000	104.997	0.092	0.088	125.000	124.383	0.033	124.383
5	5.004	338.306	0.443	0.131								
Avg.			**0.510**	**0.292**			**0.069**	**0.107**			**0.040**	**0.053**

Shape precision of pulse waveform (Amplitude [H], Time [T])												
PP (mmHg)	H1/H			H4/H			H5/H					
	Mean	SD	CV (%)	Mean	SD	CV (%)	mean	SD	CV (%)	H average (CV, %)		
40.000	0.408	0.002	0.417	0.076	0.001	1.378	0.083	0.001	1.136	**1.059**		
60.000	0.501	0.002	0.330	0.102	0.002	1.751	0.104	0.002	1.814			
80.000	0.581	0.001	0.194	0.120	0.002	1.369	0.128	0.002	1.231			
105.000	0.654	0.002	0.294	0.141	0.002	1.463	0.148	0.002	1.331			
Avg.		0.002	0.309		0.002	1.491		0.002	1.378			

PP (mmHg)	T1/T			T4/T			T5/T			T average (CV, %)		
	Mean	SD	CV (%)	Mean	SD	CV (%)	Mean	SD	CV (%)			
40.000	0.181	0.005	2.828	0.556	0.004	0.795	0.636	0.007	1.088	**1.603**		
60.000	0.178	0.005	2.681	0.552	0.005	0.872	0.626	0.013	2.128			
80.000	0.172	0.004	2.177	0.539	0.005	0.979	0.614	0.005	0.809			
105.000	0.171	0.005	2.709	0.531	0.006	1.074	0.606	0.007	1.095			
Avg.		0.005	2.599		0.005	0.930		0.008	1.280			

* SD: standard deviation, CV: coefficient of variation, H: sum of the base pressure and pulse pressure, H1: amplitude of the first peak in the pulse wave, H4: amplitude of the second valley in the pulse wave, H5: amplitude of the third peak in the pulse wave, T: the total time of one pulse wave, T1: time to the first peak in the pulse wave, T4: time to the second valley in the pulse wave, T5: time to the third peak in the pulse wave.

The coefficient of variation (CV) for each pulse wave shape was also calculated to assess the variability of the measured pulse shape. As a result, the amplitude variability was approximately 1.1%, whereas the timing variability was approximately 1.6%, as presented in Table 2.

The pulse wave range of the developed system was measured in three main categories: the systole/diastole ratio, pulse rate, and base pressure. The systole/diastole ratio ranged from 24.5% to 57.7%. The pulse rate was reproducible from 19.9 bpm to 203.9 bpm. The base pressure was reproducible within a range from − 3.2 mmHg to 116.5 mmHg. The base pressure has relatively high error rates because of the large volume of the reservoir, which was designed to support a wide base pressure range in the developed system. To achieve a wide base pressure range and precise pulse pressure control, a dual-volume control system was implemented. This system enables both a wide base pressure range and precise pulse pressure control. Consequently, the variability of the pulse pressure is minimal, with a CV of only 0.11%. The systole/diastole ratio presented an approximately 1.04% error compared with the target ratio, the pulse rate presented a 0.34% deviation from the target rate, and the base pressure (BP) presented an error of approximately 3.60% relative to the target value. Additionally, the BP is designed to be adjustable through software control, enabling a reduction in errors. The BP in the evaluation system is controlled by a motor-driven piston that advances to apply static pressure within the wrist simulation unit. Although the unadjusted BP error was approximately 3.60%, this value reflected the wide operating range tested during validation rather than control precision. When the software-based fine adjustment function was activated, the BP error was reduced to less than 0.07%, as the control program allows direct user correction via the “adjustment (button)” interface. Therefore, the BP can be reliably maintained during device evaluation, ensuring the stability of the pressure environment. The experimental results for the pulse wave range, including the systole/diastole ratio, pulse rate range, and base pressure range, are shown in Table 3.

**Table 3:** Experimental results for the pulse wave range.

No.	Pulse range					
	Target ratio (%)	Systole/diastole ratio (%)	Target rate (bpm)	Pulse rate (bpm)	Target pressure (mmHg)	Base pressure (mmHg)
1	25.000	24.452	20.000	19.934	0.000	-3.173
2	40.000	39.675	75.000	74.645	40.000	38.171
3	50.000	50.089	130.000	129.337	80.000	77.337
4	57.143	57.691	204.000	203.899	120.000	116.510
Error rate (%)		1.036		0.341		3.603

**Fig. 8:**
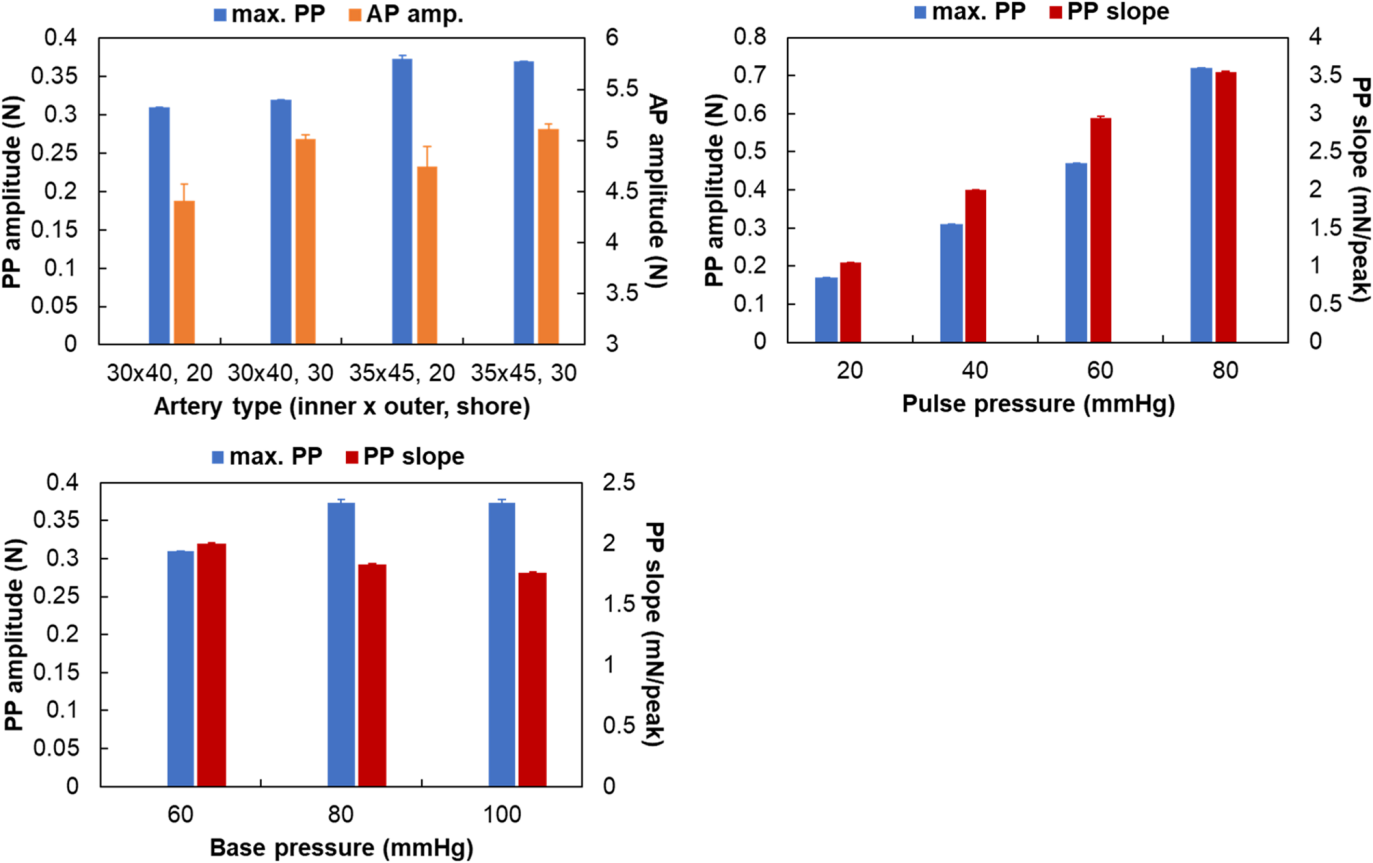
Experimental results of pulse wave characteristics based on artificial radial artery type, pulse pressure, and base pressure.

The changes in the maximum pulse pressure (max. PP) and applied pressure amplitude (AP amp.) were observed on the basis of the type of artificial radial artery. With the base pressure, pulse pressure, and pulse rate fixed at 60 mmHg, 40 mmHg, and 75 bpm, respectively, the applied pressure was gradually increased, and the pulse pressure, applied pressure at max. PP, and pulse pressure slope (PP slope) were measured. The statistical analysis revealed that arterial stiffness and size both influenced the pressure responses. Specifically, AP amp significantly increased with higher arterial stiffness (*p* < 0.001), whereas max. PP significantly increased with larger arterial diameter (*p* < 0.001). These findings indicate that vessel compliance plays a dominant role in determining applied pressure amplitude, while geometric scaling primarily affects the overall pulse pressure magnitude. When the artery was fixed at 30 × 40, 20, with a base pressure of 60 mmHg and a pulse rate of 75 bpm, the pulse pressure was varied to 20 mmHg, 40 mmHg, 60 mmHg, and 80 mmHg. The changes in pulse wave characteristics were analyzed. The results revealed that as the pulse pressure increased, both the max. PP and PP slope increased. Statistical analysis confirmed that both max. PP and PP slope exhibited significant differences across all PP levels (*P* = 20, 40, 60, 80 mmHg) with *p* < 0.001. This high sensitivity demonstrates the system’s capability to precisely evaluate devices under varying physiological pressure conditions. Finally, when the artery, PP, and pulse rate were fixed at 30 × 40, 20, 40 mmHg, and 75 bpm, respectively, while the BP was varied at 60 mmHg, 80 mmHg, and 100 mmHg, the pulse wave characteristics changed accordingly. As BP increased from 60 to 80 mmHg, max. PP showed a statistically significant increase (*p* < 0.001), but it remained constant beyond 80 mmHg, indicating that the system maintains stable measurement performance above a certain pressure range. Conversely, PP slope exhibited a statistically significant decrease with increasing BP (*p* < 0.001), suggesting that this parameter reflects the damping effect of arterial elasticity under higher preload conditions, as shown in Fig. 8. Overall, changes in pulse pressure had a greater effect on the max. PP and PP slope than changes in base pressure did. Additionally, the influences of base pressure changes and artery size changes were similar. The stiffness of the artery was found to be potentially distinguishable by the AP amp.

## Discussion

Tonometry pulse wave measurement devices require an applied pressure during measurement. Therefore, to develop a system for evaluating tonometry-based pulse wave measurement devices, a device capable of assessing the applied pressure is essential. The developed system is embedded with a force sensor for evaluating the applied pressure and incorporates a precision applied pressure system for tonometry sensor evaluation. By controlling the motor speed, the system was able to achieve an applied pressure precision of approximately 0.29%. The applied pressure measurement sensor in the developed system can theoretically measure up to 0.064 mmHg, making it a system capable of highly precise applied pressure measurements. Although considerable research has been conducted on applanation pulse wave measurement devices^15–18^, few commercialized products exist. Recently, however, interest in smartwatches, digital healthcare devices, and wearable devices for personal health management has increased. As a result, tonometry-based pulse wave measurement devices, which can provide various pulse wave indices, are expected to gain prominence. Therefore, further research into tonometry pulse wave measurement devices and evaluation systems for tonometry devices is necessary.

To minimize variability and ensure consistent validation of the system’s precision and reproducibility, a single artificial skin type was employed in this study. To make artificial skin on the radial artery, the skin thickness was determined to be approximately 1.5 mm, referencing the studies of Lee^19^, Wei^20^, and Oltulu^21^. The Young’s modulus of the skin was estimated using the methods of Wakhlu^22^, Wei^20^, and Park^23^, resulting in a skin modulus of approximately 10.2 kPa and a subcutaneous skin modulus of approximately 2.75 kPa. To replicate the thickness and Young’s modulus of artificial skin, Ecoflex™ 00–30 (skin layer) and Ecoflex™ 00–10 (subcutaneous layer; Smooth-On, Inc., PA, USA) were used to fabricate artificial skin with a 1.5 mm skin layer and a 10 mm subcutaneous layer. However, to understand the impact of artificial skin on pulse wave measurement, it is necessary to fabricate various types of artificial skin with different forms and elasticities and evaluate their influence through experiments. The representative physiological properties of human skin and arteries referenced for this study are summarized in Supplementary Table S2, which provides comparative data on skin thickness, elasticity, vessel diameter, wall thickness, and modulus values reported in the literature. The size and elasticity of the artificial radial artery were designed based on studies by Avolio^24^, Laurent^25^, Getachew^26^, Chamiot-Clerc^27^, and Leguy^28^. Due to the challenges of small-scale production and fabrication constraints, we were only able to produce four types of artificial arteries with two sizes and two stiffness levels. Therefore, the evaluation of the proposed system was conducted using these four artery types. Artificial radial arteries were fabricated with inner diameters of 3.0 mm and 3.5 mm, a thickness of 0.5 mm, and elastic moduli of 0.34 MPa (Shore A 20) and 0.59 MPa (Shore A 30). According to studies on vascular elasticity, the measured elastic modulus varies widely, ranging from approximately 0.3 MPa to 3 MPa, depending on the measurement method (tensile, indentation, or compression) and direction (axial or radial), making it challenging to determine an exact value. While the present study employed a single optimized artificial skin and four artery types to reduce experimental complexity, it is acknowledged that achieving a more comprehensive representation of physiological variability requires the development of new fabrication methods. In future work, we plan to expand the diversity of artificial tissues by introducing multi-layer composite modeling, variable-thickness molding, and advanced elastomer blending techniques to achieve a wider range of mechanical properties. Such improvements will allow statistical comparisons across different skin and vessel types, supporting a more robust analysis of how tissue variability influences pulse wave measurement accuracy. Finally, the primary objective of this study was to establish a precise and reliable system for evaluating tonometry-based pulse wave measurement devices. Therefore, the influence of skin and arterial variability was intentionally minimized during initial validation. Nevertheless, future studies will focus on quantifying the impact of tissue heterogeneity on pulse wave formation and transmission, providing deeper insight into the physiological realism and diagnostic reliability of the proposed evaluation system.

Although some tonometry pulse wave measurement devices are available on the market, most of them only provide results without offering raw data, making it difficult to evaluate the devices effectively. While previous studies reported that the AIx values of other arterial applanation tonometry devices, such as the SphygmoCor (Cardiex, Sydney, Australia), had a standard deviation (SD) of 3–8% for intervisit reproducibility^29–31^, these systems cannot evaluate applied pressure, pulse pressure, or pulse rate directly via the developed system. Therefore, tests were conducted using a previously developed pulse sensor^15^ to evaluate whether it could assess the applied pressure, pulse pressure, and pulse rate. The results revealed that the coefficient of determination (r²) for applied pressure was 0.9956 in the range of 25 to 250 mmHg, the r2 for pulse pressure was 0.9946 in the range of 40 to 120 mmHg, and the r2 for pulse rate was 0.9985 in the range of 20 to 150 bpm. While these results demonstrate relatively high accuracy, there are limitations, as the tests were conducted using a prototype sensor rather than a commercialized device. In the future, we plan to test the practicality of the system via devices such as the SphygmoCor, HEM-9000AI (Omron, Japan), T-Line (Tensys Medical Inc., CA, USA), BPro Evo (Healthstats, Singapore), PST-301 (Millar, Inc., TX, USA), and DMP-LIFE PLUS or DMP-LIFE (DAEYOMEDI Co., Ltd., Korea) devices.

To further validate the developed evaluation system, its performance specifications were compared with the international standard ISO 18615:2020, which defines the general performance requirements for electric radial pulse tonometric devices. Additionally, a comparative assessment was conducted using the Harvard Apparatus Pulsatile Blood Pump (Model 1421), a widely recognized reference simulator in hemodynamic research. The detailed comparison results, summarized in Supplementary Table S1, demonstrate that the proposed system not only satisfies but exceeds the ISO 18,615 performance thresholds. Furthermore, when compared with the Harvard Apparatus simulator, the developed system provides a broader operational range and superior precision in controlling applied and pulse pressures. These findings confirm the system’s potential as a standardized and high-precision evaluation platform for tonometry-based pulse wave measurement devices.

The evaluation system developed in this study was designed specifically to assess tonometry-based pulse wave measurement devices that detect arterial pulsation of the radial artery through mechanical applanation. To reproduce these conditions accurately, the system employs an air-based pulse wave generation mechanism capable of precise control of applied and pulse pressures. This configuration provides high repeatability and fidelity in mechanical pulse wave reproduction; however, it does not allow the generation of optical signals required for photoplethysmography (PPG) measurement. Nevertheless, the system architecture has been designed with expandability toward a fluid-based pulse wave generation module. The use of a fluidic medium would enable more realistic simulation of vascular dynamics and light–tissue interactions, making it feasible to evaluate PPG-based devices in the future. Transitioning to a fluid-based evaluation system requires further investigation, as various factors such as fluid viscosity, color, flow characteristics, and the optical transmittance of artificial skin and vessel materials must be carefully considered. Future research will focus on refining these parameters to establish a unified and comprehensive platform applicable to both tonometry- and PPG-based device evaluations.

## Conclusions

We developed a novel evaluation system for tonometry-based pulse wave measurement devices by integrating a pressing and fixation unit, a system control and drive unit, a wrist simulation unit, and a 3D cam-based pulse wave generation unit. The system demonstrated high reproducibility in simulating physiological pulse waves under varying base and pulse pressures and enabled analysis of the effects of artificial radial artery properties on measurement performance. Experimental validation showed excellent precision, with mean coefficients of variation (CVs) of 0.29% for applied pressure, 0.11% for pulse waves, and 0.05% for pulse rate. For pulse wave shape evaluation, the CVs of parameters H and T were 1.06% and 1.60%, respectively. The system also covered a wide physiological range, with the systole/diastole ratio (SDR) spanning 24.45% to 57.69% (error ratio 1.04%), pulse rate ranging from 19.93 bpm to 204.90 bpm (error ratio 0.34%), and base pressure ranging from − 3.17 mmHg to 116.51 mmHg (error ratio 3.60%). By providing a reliable and physiologically realistic platform for standardized assessment, this system is expected to support the development and calibration of tonometry-based and wearable pulse wave measurement devices. Clinically, it can contribute to improving the reliability of cardiovascular monitoring, while from an industrial and research perspective, it offers a valuable benchmark for device validation and advances the reproducible generation and analysis of radial artery pulse waves.

## Supplementary Information

Below is the link to the electronic supplementary material.

Supplementary Material 1

## Data Availability

The datasets generated and/or analyzed during the current study are available from the corresponding author on reasonable request.
