# Supplementary material for: Development of a high-precision evaluation system for radial pulse wave applanation tonometry devices
Source: Sci Rep. 2026 Mar 31;16:15598. doi: 10.1038/s41598-026-45661-4 (PMC13187050; doi:10.1038/s41598-026-45661-4)
Supplement: Supplementary file 1 — Supplementary Material 1 [file 41598_2026_45661_MOESM1_ESM.docx]

**Supplementary Material**

Figure S1


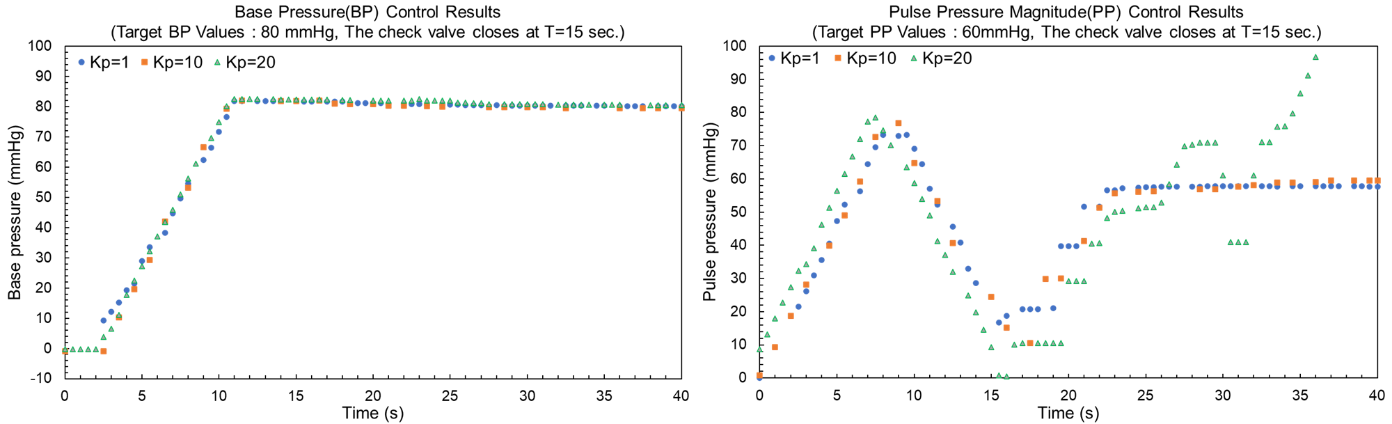


(a) (b)

Figure S1. The Kp-specific control results of the base pressure (a) and pulse pressure magnitudes (b) obtained by the proposed dual-volume control system.

As shown in Figure S1 (a), the base pressure feedback control algorithms operate until the error reaches within ±0.5% of the target value, 80mmHg. Figure S1 (b) shows that proper tunning of the proportional gain values is important for both system stability and performance. Excessively high proportional gains can make the system unstable, as shown in the plot at Kp2=20 in Figure S1 (b).

Figure S2


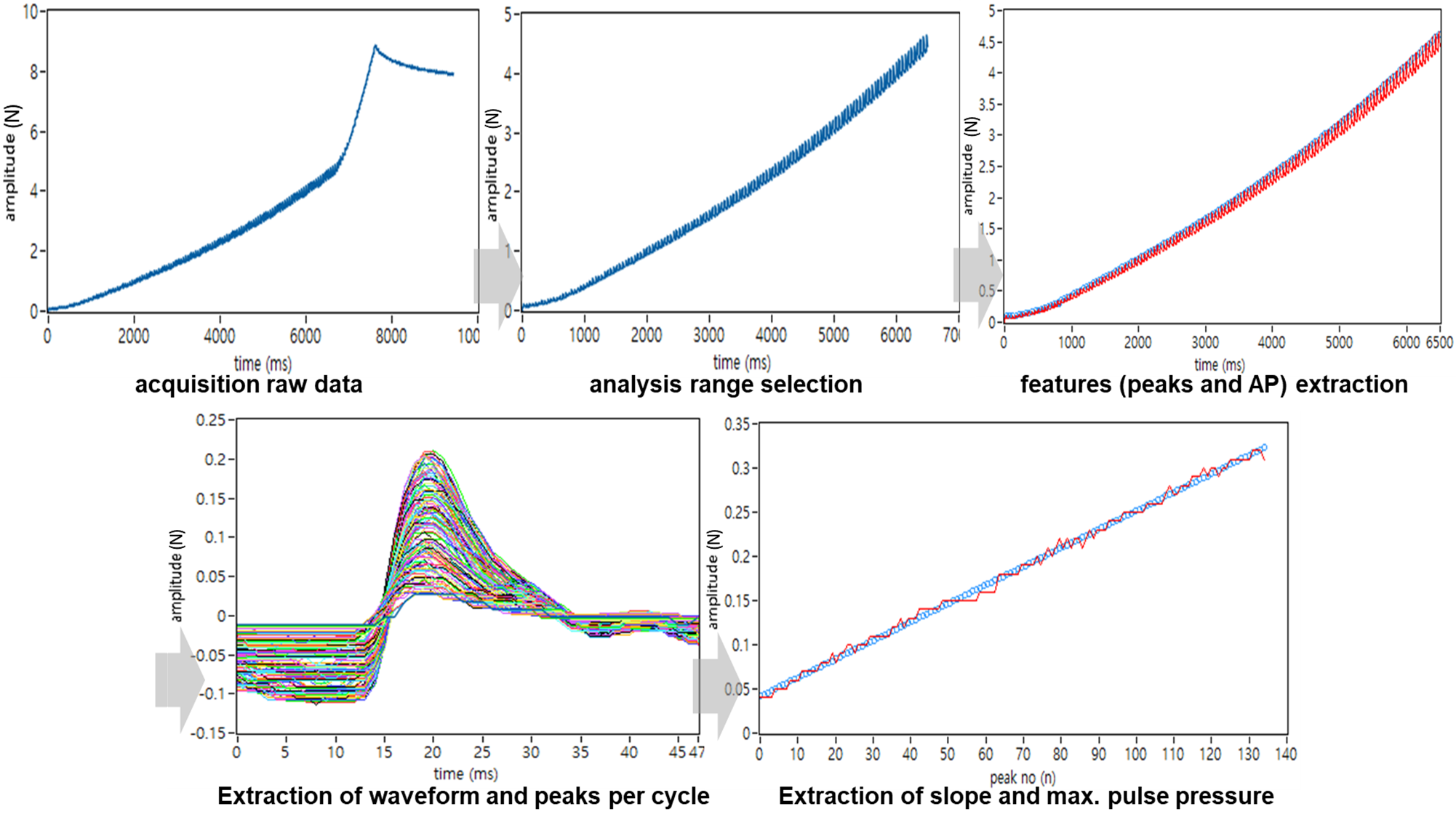


Figure S2. Methods for range selection, feature extraction, waveform and peak value detection for a cycle, and pulse peak slope and maximum amplitude detection from acquired data to evaluate variability in pulse wave measurement on the basis of artificial radial artery characteristics (raw data → analysis section selection → peak and AP extraction → waveform superimposition by a pulse period → extraction of slope and maximum amplitude of pulse wave to applied pressure).

Table S1

*Table S1. Performance comparison among the developed evaluation system, ISO 18615:2020 standard requirements, and the Harvard Apparatus Pulsatile Blood Pump*

| Parameter | ISO 18615 Requirement | Proposed Evaluation System | Harvard Apparatus Pulsatile Blood Pump (Model 1421) |
| --- | --- | --- | --- |
| Applied Pressure (AP) | 0–120 mmHg  (±6 mmHg / ±5%) | 0–338 mmHg  (SD: ±0.5 mmHg / CV: 0.29%) | Not applicable |
| Pulse Pressure (PP) | 0–105 mmHg  (±5 mmHg / ±5%) | 0–105 mmHg  (SD: ±0.07 mmHg / CV: 0.11%) | Not specified  (stroke volume: 4–30 mL) |
| Pulse Rate (PR) | 40–150 bpm  (±5 bpm) | 20–204 bpm  (SD: ±0.04 bpm / CV: 0.05%) | 20–200 bpm |
| Systole/Diastole Ratio (SDR) | — | 24–58%  (error ratio: 1.04%) | 35–50%  (adjustable) |

Table S2

*Table S2. Summary of reported physiological properties of human skin and radial artery*

| **Category** | **Reference Parameter** | **Clinical Values and Sources** |
| --- | --- | --- |
| **Skin Properties** | **Skin Thickness** | • Epidermis + Dermis (μm): Front of arm: 1,012.6 ± 233.3, Back of arm: 1,171.8 ± 379.3 [19]  • Various sites: Viable epidermis: 31–637 (μm), Dermis: 521–1,977 (μm) [20] • Total skin thickness (μm): Front of arm: 1,460 ± 260  [Hesselstrand, Roger, et al. "High-frequency ultrasound of skin involvement in systemic sclerosis reflects oedema, extension and severity in early disease." Rheumatology 47.1 (2008): 84-87.] |
|  | **Skin Elasticity** | • Young’s modulus (Elastography): Forearm: 37.2 kPa, Hand: 34.5 kPa [22]  • Mean elastic modulus (Human): Human: 50.26 ± 19.3 kPa, Human (*in vivo*): 108.19 ± 140.3 [20]  • Skin Modulus: 30 years: 10.2 kPa, 60 years: 7.5 kPa, 80 years: 5.3 kPa  [Zahouani, Ha, et al. "Characterization of the mechanical properties of a dermal equivalent compared with human skin in vivo by indentation and static friction tests." Skin research and technology 15.1 (2009): 68-76.] |
| **Vascular Properties** | **Vessel Diameter** (Internal) | • 0.32 cm [24]  • Right radial artery: 3.79 ± 1.44 mm, Left radial artery: 3.79 ± 1.78 mm [26] |
|  | **Vessel Thickness** | • 0.43 mm [24]  • Mean: 0.37 ± 0.22 mm, Right & Left radial: 0.34 ± 0.23 mm [26] |
|  | **Vessel Elasticity** (Modulus) | • 8 × 10^6 dyn/cm^2 (=0.8 MPa) [24]  • 0.3–1.0 MPa (at Elastin part)  [Camasão, et. al. "The mechanical characterization of blood vessels and their substitutes in the continuous quest for physiological-relevant performances. A critical review." Materials Today Bio 10 (2021): 100106.] |

Table A1

*Table A1. Comparison of base pressure before and after software adjustment*

| target pressure (mmHg) | before adjustment (mmHg) | after adjustment (mmHg) |
| --- | --- | --- |
| 0 | -3.173 | 0.170 |
| 40 | 38.171 | 40.047 |
| 80 | 77.337 | 79.967 |
| 120 | 116.51 | 119.941 |
| error rate (%) | 3.603 | 0.069 |
